# The Nature of Iron Deposits Differs between Symptomatic and Asymptomatic Carotid Atherosclerotic Plaques

**DOI:** 10.1371/journal.pone.0143138

**Published:** 2015-11-25

**Authors:** David Kopriva, Anastasye Kisheev, Deiter Meena, Shaneen Pelle, Max Karnitsky, Andrea Lavoie, Josef Buttigieg

**Affiliations:** 1 Department of Surgery (Vascular Surgery), Regina Qu’Appelle Health Region, College of Medicine, University of Saskatchewan, Regina, Canada; 2 Department of Biology, University of Regina, Regina, Canada; 3 College of Medicine, University of Saskatchewan, Regina, Canada; 4 Department of Medicine (Cardiology), Regina Qu’Appelle Health Region, College of medicine, University of Saskatchewan, Regina, Canada; Monash University, AUSTRALIA

## Abstract

Iron within atherosclerotic plaque has been implicated as a catalyst of oxidative stress that causes progression of plaque, and plaque rupture. Iron is believed to accumulate within plaque by incorporation of erythrocytes following plaque rupture and hemorrhage. There is only indirect evidence to support this hypothesis. Plaque specimens were obtained from ten symptomatic and fifteen asymptomatic patients undergoing carotid endarterectomy at a single institution. Plaques were sectioned for study using synchrotron radiation induced X-ray fluorescence the study the distribution of zinc, calcium and iron. Histologic staining was carried out with Prussian Blue, and immunohistochemical staining was done to localize macrophages with CD68. Data were compared against patient clinical variables. Ten symptomatic (15 ± 10 days between index symptoms and surgery) and fifteen asymptomatic carotid plaques were studied. Zinc and calcium co-localized in mineralized areas of symptomatic and asymptomatic plaque. Iron was identified away from zinc and calcium in both symptomatic and asymptomatic plaques. Within the symptomatic plaques, iron was found within the thrombus associated with plaque rupture and hemorrhage. It did not stain with Prussian Blue, but was found in association with CD68 positive macrophages. In symptomatic plaques, the abundance of iron showed an association with the source patient’s LDL cholesterol (R^2^ = 0.39, Significance F = 0.05). Iron in asymptomatic plaque was present as hemosiderin/ferritin that stained positive with Prussian Blue, and was observed in association with CD68 positive macrophages. Iron in acutely symptomatic plaques is found within thrombus, in the presence of macrophages. The abundance of iron in symptomatic plaques is associated with the source patient’s LDL cholesterol. Within asymptomatic plaques, iron is found in association with macrophages, as hemosiderin/ferritin.

## Introduction

Atherosclerotic plaque at the carotid artery bifurcation is an important cause of stroke. This is the basis of plaque removal (carotid endarterectomy) as a useful stroke reduction strategy [1–7]. Plaque accumulation is a slow process that occurs over a period of years or decades, under the influence of well-known risk factors such as advancing age, smoking, diabetes, hypertension and elevated LDL cholesterol. Stroke symptoms occur when carotid bifurcation plaque ruptures and forms thrombus, causing material to embolize into the cerebral circulation [7–10].

Oxidation of LDL cholesterol is important throughout the process of atherosclerotic plaque formation and destabilization [11]. Iron is found at high concentrations in the walls of atherosclerotic arteries [12,13] and accumulates mainly in macrophages [14]. The oxidative process is proposed to occur through Haber-Weiss and Fenton reactions within plaque macrophages [15–18].

It has been assumed that iron enters plaque macrophages through the endocytosis of heme rich erythrocytes following plaque rupture, hemorrhage, and healing. This assumption is based on previous work, in coronary arteries, demonstrating that the progression of atherosclerotic plaques is associated with histologic evidence of plaque rupture and healing, through the deposition of new connective tissue [19–21]. Similar healing of unstable plaque has been demonstrated in carotid arteries [22]. In-vitro studies have shown the ability of macrophages to ingest erythrocytes [23], and phagocytosed erythrocytes have been identified in plaque macrophages near intra-plaque microvessels, suggesting previous hemorrhage into the plaque[24].

We undertook the present work in order to seek further evidence that atherosclerotic plaques at the carotid bifurcation accumulate iron through rupture, hemorrhage and healing.

## Materials and Methods

### Patient Enrolment and Surgery

Twenty-five patients undergoing carotid endarterectomy for stroke prevention were enrolled. Patients’ provided their written consent, which was then saved on file. The protocol for this study was reviewed by the Biomedical Research Ethics Board of the University of Saskatchewan and approved by the University of Saskatchewan, Regina Qu’Appelle Health Region, and the University of Regina through a harmonized ethics review process.

All patients had carotid bifurcation atherosclerosis. Operative indications included either symptomatic or asymptomatic carotid artery plaque. Symptomatic carotid artery plaque was defined by the presence of focal neurologic symptoms attributable to the index carotid lesion, associated with > 50% stenosis of the internal carotid artery origin. Symptoms could include ipsilateral transient monocular vison loss (amaurosis fugax), contralateral sensory or motor loss, or aphasia for left sided carotid lesions. Symptoms could be transient in nature, or fixed, causing a permanent neurologic defect. The onset of symptoms must have occurred within 120 days prior to surgery, in accordance with NASCET definitions [1]. Asymptomatic carotid plaque was defined by the presence of atherosclerosis causing > 80% stenosis at the origin of the internal carotid artery, without ipsilateral neurologic symptoms, in a patient with low surgical risk and > 2 years predicted longevity as assessed by the patient’s surgeon. The degree of carotid stenosis was assessed by NASCET criteria [1]. Quantification of internal carotid artery stenosis was done with duplex ultrasound, CT angiography, or both. All patients were recruited through a single, three surgeon vascular surgery practice between April 2012 and April 2015. Patients who provided informed consent to undergo carotid endarterectomy for stroke prevention were recruited to participate in this study.

After signed, informed consent to participate was provided, the following clinical data were collected regarding each subject: age, gender, side of the index carotid lesion, ethnicity of the patient, current medications, total cholesterol, LDL cholesterol, HDL cholesterol, serum triglyceride levels, blood pressure, presence or absence of diabetes, date of the last ipsilateral focal neurologic symptom, and the date of carotid endarterectomy. In addition, subjects were asked about their smoking status and were categorized as never having smoked, being current smokers, or being past smokers who quit. The duration of smoking cessation was recorded.

Carotid endarterectomy was performed according to the standard practice of the subject’s surgeon with respect to the use of general or local anesthesia, use of intraoperative shunt, and patching. Once the carotid plaque was removed, it was placed in 10% neutral buffered formalin (Leica Biosystems, Winnipeg, Canada) and stored at 4 degrees C until further processing.

### Tissue Preparation

The carotid plaques were then embedded in optimal cutting temperature embedding medium (OCT) in metallic cryomolds (25mm X 25mm X 15mm). Using a cryostat (MICROM HM 550) with a Teflon coated blade at -20 degrees C, 10μm thick sections of carotid artery tissue were sequentially obtained through the entire plaque. Tissue sections were alternately mounted on glass microscope slides and metal free Thermanox plastic cover slips (Fisher Scientific, Waltham, MA). The tissue was air dried at room temperature for an hour then placed in a desiccator. The tissue sections were sequentially numbered and labelled.

The preparation of specimens with alternating glass and Thermanox slides was necessary because glass microscope slides contain trace metals that preclude synchrotron imaging of the elements of interest in this study. Thermanox slides are depleted in the elements of interest, making them a good choice for synchrotron imaging. Alternating sections were mounted on glass microscope slides to facilitate immunohistochemical staining, for which Thermanox is unsuitable due to its autofluorescence in the 470–550 nm range.

### Synchrotron Imaging

The Thermanox mounted tissue sections were visually examined and the samples which appeared to be from the most active part of each carotid plaque, based on plaque ulceration or degree of stenosis, were selected for synchrotron imaging. Due to limitations on access to synchrotron beam time, the samples were imaged in batches. All symptomatic carotid plaques, as well as five asymptomatic plaques were imaged at Stanford Synchrotron Radiation Lightsource (SSRL) using X-ray fluorescence at an energy of 13.45 KeV, with a resolution of 40μm and a dwell time of 200ms per point. In addition to the elements of interest (zinc, calcium, and iron), fluorescence counts were obtained for copper, sulfur, potassium, titanium, manganese, nickel, and arsenic. Ten asymptomatic plaques, as well as four of the five asymptomatic plaques previously imaged at SSRL, were studied at the Canadian Light Source (CLS) synchrotron facility VESPERS beam line, using pink beam and X-ray fluorescence. Element maps were generated at 80μm resolution and a dwell time of 500ms per point. A subset of six of the asymptomatic plaques were restudied at CLS with 5μm resolution scans of localized iron rich areas. Digital data for each element were stored in a separate data channel.

Data were collected in electronic files and processed using Sam’s Microprobe Analysis Kit (Sam Webb, Stanford Synchrotron Radiation Laboratory). Fluorescence maps were produced for each element; each data channel was corrected for variations in incident beam strength and detector deadtime. Fluorescence channels for all elements were then combined into a single channel which was processed using inverse binary thresholding. Threshold levels were selected to maximize the signal from the plaque while reducing noise from empty areas around the plaque and within the plaque lumen. The threshold processed channels were used to create a digital mask. The mask was then applied to each data channel to eliminate noise from peripheral areas of the slide. Fluorescence levels for each element were quantified. Element correlation plots were obtained. Statistical analysis was done using Excel (Microsoft Corporation). Categorical variables were compared using Fisher’s exact test and continuous variables with Student’s t test. Linear regression was used to study correlations between continuous variables and ANOVA to study correlations with categorical variables. Ninety-five percent confidence intervals were determined, and p<0.05 was considered statistically significant.

### Prussian Blue Stain

In order to further characterize the nature of iron deposits identified on synchrotron imaging, we performed staining with Prussian Blue (PB). PB stains hemosiderin and, to a lesser extent, ferritin deposits. The stain consists of equal quantities of 20% hydrochloric acid (v/v) and 10% potassium ferrocyanide (w/v, Sigma). Glass mounted sections were stained with PB for 20 min, washed in running water for 1 min, counterstained with 0.5% aqueous nuclear fast red solution (Sigma) for 1 min. Subsequent dehydration steps used exact timings in order to maintain consistent counterstain intensity and distribution.

The glass mounted carotid plaque section from the location adjacent to each synchrotron imaged specimen was stained with PB and surveyed in 100 μm segments. PB staining was scored from 0 (no blue present) to 4 (dark blue present).

### Immunohistochemical Staining

Sections mounted on glass slides, a maximum of 60 μm distant from sections imaged by synchrotron underwent immunohistological staining. Sections were re-hydrated with 1x PBS for 15 minutes. CD68 (abcam 1250212, 1:600, host species rabbit) was added to a blocking solution consisting of 0.3% Glycine, 1x PBS, 1% BSA, 0.1% Triton X-100 and incubated with the specimen overnight at 4°C. The next day, sections were washed 3 x (5 min each) with PBS. Secondary antibody was added (alexa 647 goat anti-rabbit; abcam 150091) for 45 minutes. After incubation period, sections were washed 3 x in PBS and flurogold mounting media was added. As a control, we omitted the primary antibody, to confirm secondary antibody specificity.

## Results and Discussion

Twenty-five patients were enrolled in the study: ten with symptomatic carotid stenosis and fifteen with asymptomatic stenosis. Subjects ranged in age from 45 to 81 years. (mean age 69.8 ± 3.5 years). The mean ages of the symptomatic and asymptomatic patients were not different (p = 0.46). Two of fifteen asymptomatic patients were female (13%), while four of ten symptomatic patients were female (40%; p = 0.18). Symptomatic subjects underwent carotid endarterectomy at a mean of 15 ± 10 days (range 5–56 days) following their last ipsilateral carotid territory neurologic event. Two asymptomatic patients had a history of remote, ipsilateral carotid territory symptoms 30 years, and 125 days, respectively and thirteen had no history of ipsilateral neurologic symptoms at any time in the past. Three of ten symptomatic patients were diabetic (30%) whilst seven of fifteen (47%) asymptomatic patients were diabetic (p = 0.68). The mean systolic and mean diastolic blood pressures were similar in the symptomatic and asymptomatic subjects (p = 0.73 and p = 0.35, respectively). Of the symptomatic subjects, one had never smoked, one was a current smoker, and eight had quit smoking 1–26 years prior to surgery (mean quitting duration 11.9 years). Of the asymptomatic subjects, six had never smoked, two were current smokers, and seven had quit smoking between four months and 44 years prior to surgery (mean quitting duration 14.5 years; p = 0.72).

### Synchrotron Imaging of Carotid Plaques

We noted only low levels of fluorescence from manganese, copper, nickel, arsenic and titanium in our samples. There was no correlation between the intensity of X-ray fluorescence from any of these elements and the symptomatic status of the subject.

**Fig 1:**
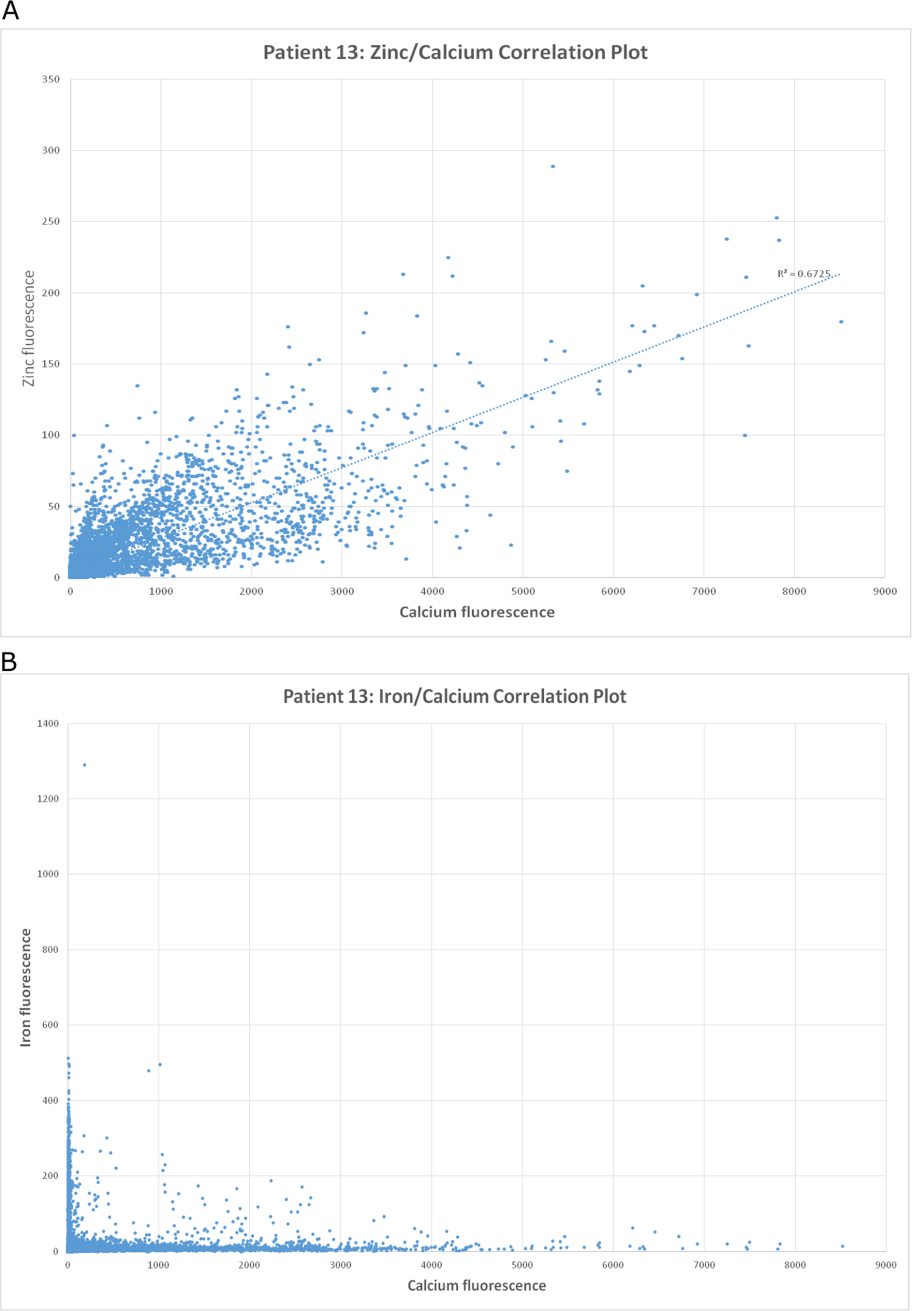
Synchrotron radiation correlation plot. (A) A correlation plot, obtained by synchrotron induced X-ray fluorescence, demonstrating zinc fluorescence as a function of calcium fluorescence at each data point obtained by scanning a single, typical asymptomatic atherosclerotic plaque. This graph demonstrates that zinc and calcium tend to co-localize. (B) A correlation plot, from the same sample shown in (A), demonstrating iron fluorescence as a function of calcium fluorescence at each data point obtained by scanning a single, typical asymptomatic atherosclerotic plaque. This graph demonstrates that iron tends to localize away from calcium rich areas of plaque.

Plaques showed variable amounts of calcium and zinc fluorescence, depending on the degree of plaque mineralization. However, these elements consistently showed co-localization in areas of mineralized plaque (Fig 1A), along with sulfur and potassium fluorescence. There was no association between the symptomatic status of a plaque and the amount of calcium, zinc, potassium or sulfur fluorescence observed. Within the group of symptomatic plaques, there was no statistically significant difference in the amount of calcium fluorescence observed between plaques from diabetics, compared with plaques from non-diabetics (p = 0.85). However, within the group of asymptomatic plaques, we observed significantly more calcium fluorescence in plaques from diabetics, compared with non-diabetics (p = 0.03).

**Fig 2:**
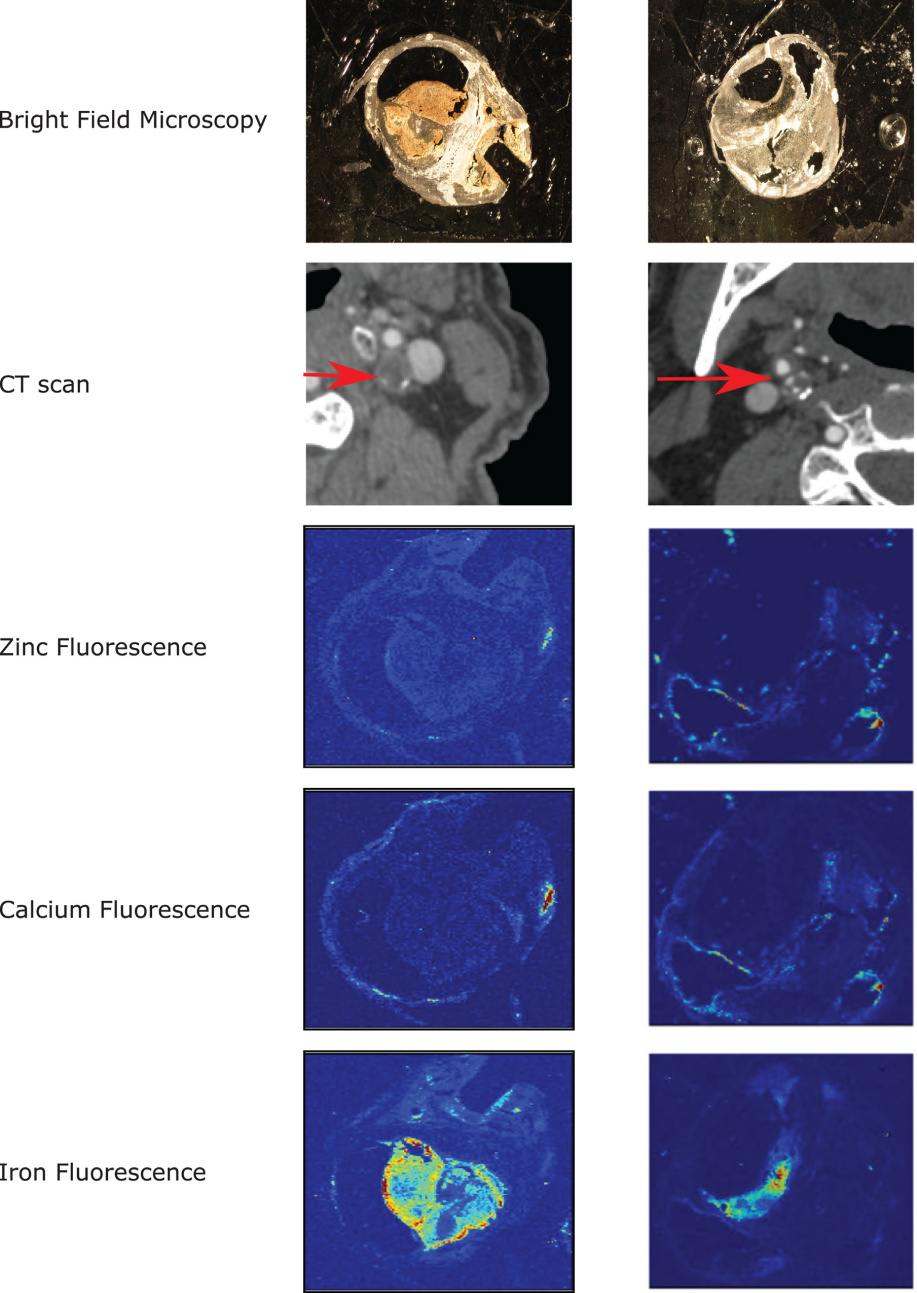
Zinc localization. A typical example of a symptomatic (five days after the onset of neurologic symptoms) and an asymptomatic carotid plaque are shown. (A) and (B) show each specimen under bright field microscopy. (C) and (D) demonstrate the appearance of approximately the same area of plaque, in the same orientation, on preoperative CT scanning (red arrow indicates the carotid plaque). (E) and (F) demonstrate zinc X-ray fluorescence of the specimen induced by synchrotron radiation. (G) and (H) demonstrate calcium X-ray fluorescence. (I) and (J) demonstrate iron fluorescence.

**Fig 3:**
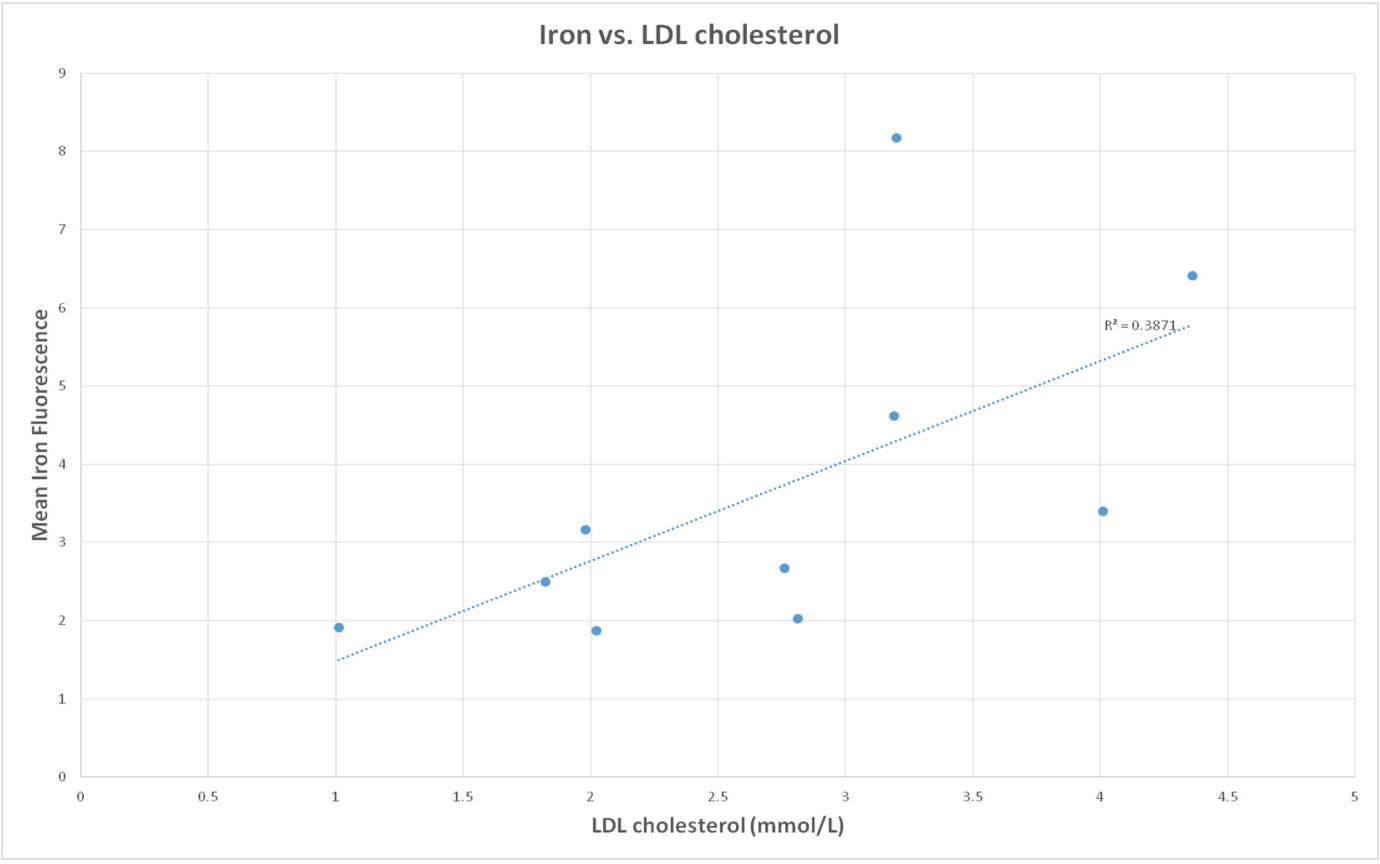
Relationship between iron deposits and LDL cholesterol. The mean iron fluorescence within each symptomatic plaque is show against the source patient’s LDL cholesterol.

Iron fluorescence was observed to occur away from zones of mineralization (Fig 1B), in sub-intimal locations of both symptomatic and asymptomatic carotid plaques (Fig 2). Within the group of symptomatic plaques, iron fluorescence was associated with areas of plaque rupture and hemorrhagic transformation. The source of iron in asymptomatic plaques could not be determined with synchrotron imaging. There was no statistically significant difference in the intensity of iron fluorescence in symptomatic plaques compared with asymptomatic plaques (p = 0.12). Similarly there were no significant associations between the intensity of iron fluorescence and patient age, the presence of diabetes, smoking status, or blood pressure. Within the group of symptomatic plaques, an association was observed between increasing iron fluorescence intensity and elevated LDL cholesterol in the source patient (Fig 3; R^2^ = 0.39; significance F = 0.05). This association was not observed in asymptomatic carotid plaques.

### Prussian Blue (PB) and Immunohistochemical Staining

**Fig 4:**
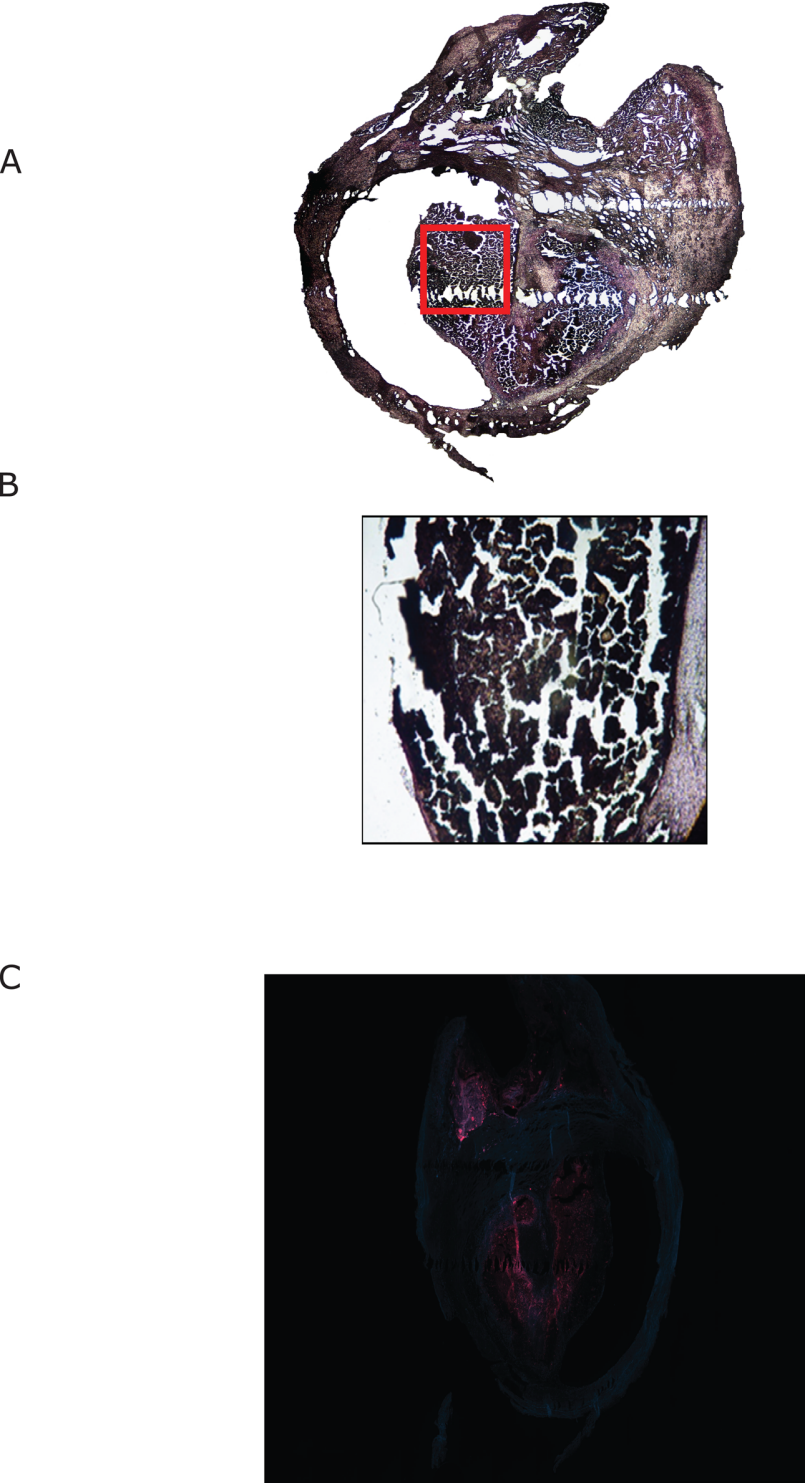
Iron deposition in symptomatic plaques. The symptomatic plaque shown in Fig 2 is demonstrated, stained with Prussian Blue (A). The red box in (A) is enlarged in (B) and demonstrates thrombus. (C) demonstrates a nearby section of the same plaque stained for macrophage specific CD68 (in red).

Within the group of symptomatic carotid plaques, the areas identified by synchrotron imaging to have a strong iron signal were found to be associated with thrombotic material that did not take up PB stain (Fig 4). The PB stain score within the group of symptomatic plaques was zero. Sections from a series of five symptomatic plaques were stained at 100µm intervals from the center of the plaque to the ends. No areas of PB stain uptake were identified away from the central area of each plaque.

**Fig 5:**
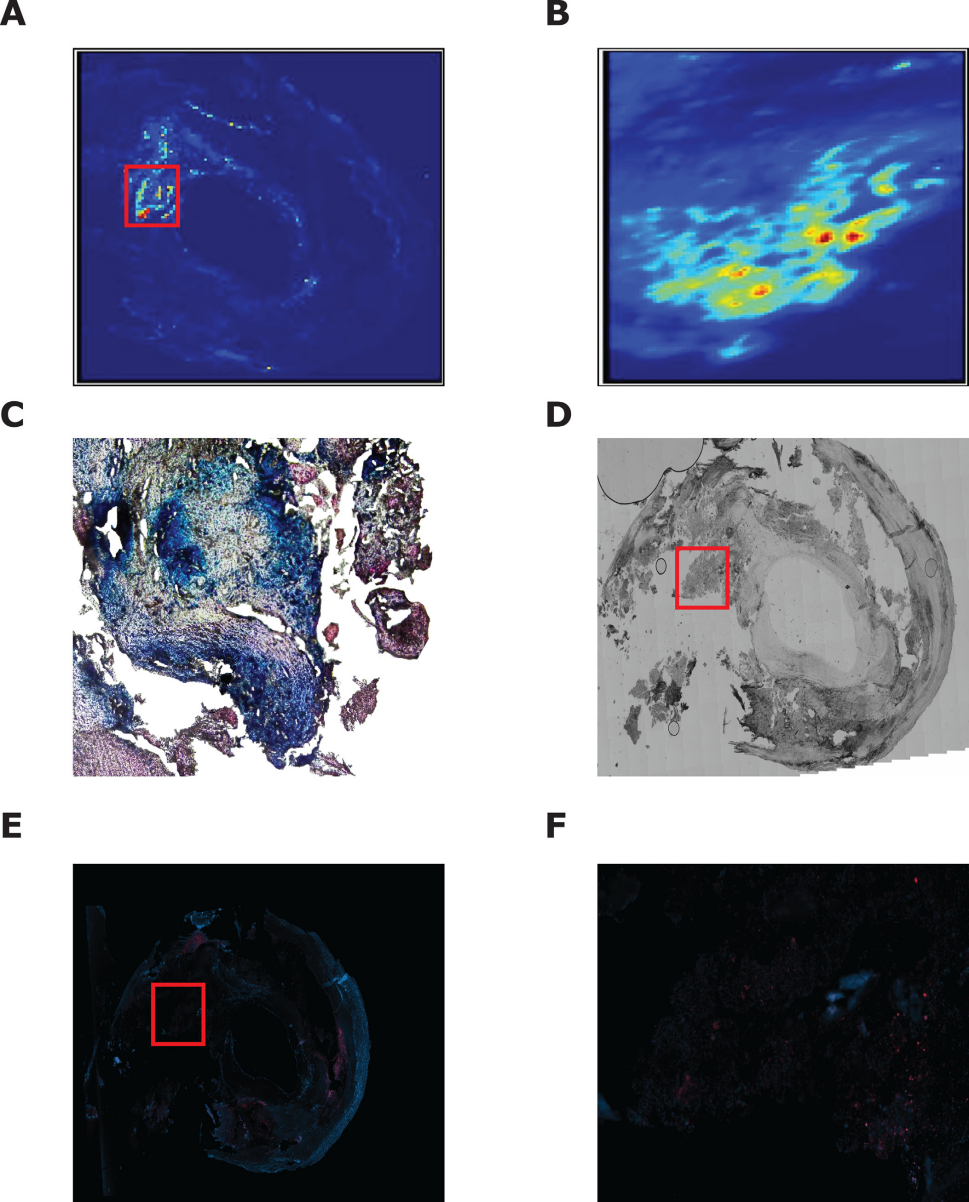
Iron deposition in asymptomatic plaques. A typical asymptomatic plaque is demonstrated showing (A) synchrotron induced iron X-ray fluorescence. (B) shows a high resolution iron X-ray fluorescence scan (5µm resolution– 415 x 680 μm scan area) of the area shown by the white box in (A). (C) shows the same area of the same specimen stained with Prussian Blue. (D) A bright field photomicrograph of a plaque section taken from 60µm away from the sample shown in (A). (E) shows CD68 staining of the specimen in (D). CD68 positive areas are seen in red. (F) is the high power field showing the corresponding area in (C) stained with CD68 (in red).

In contrast to symptomatic carotid plaques, asymptomatic plaques did not contain thrombotic material. Areas of intense iron fluorescence were associated with PB stain uptake (Fig 5), consistent with the presence of hemosiderin or ferritin. The mean intensity of PB staining within asymptomatic carotid plaques was 2.8 ± 0.4.Sections taken through asymptomatic plaques at 100µm intervals demonstrated other deposits of PB uptake, up to 800µm from the plaque center.

Immunohistochemical staining for CD68, a macrophage specific antigen, was performed on glass slide mounted sections of plaque taken no further than 60µm from the corresponding sample imaged with synchrotron X-ray fluorescence mapping. There was close association of CD68 staining macrophages, both with PB stain uptake in asymptomatic plaques, and areas of iron fluorescence in both symptomatic and asymptomatic carotid plaques. The thrombotic material observed as the source of iron fluorescence in symptomatic plaques was found to be associated with intense localization of macrophages. Within the set of asymptomatic plaques, the iron fluorescence observed with synchrotron mapping was seen to localize with PB positive hemosiderin or ferritin, in the presence of CD68 staining macrophages.

### Iron Localization in Atherosclerotic Plaques

Iron has been presumed to accumulate within atherosclerotic plaques through a series of steps beginning with plaque rupture and hemorrhage. Macrophages are stimulated by hemoglobin exposure to differentiate into M2 polarized, mannose receptor positive macrophages that are specialized for phagocytosis [25,26]. Endocytosis of erythrocytes by this macrophage sub-population then leads to conversion of heme iron into ferritin, hemosiderin and transferrin, possibly recycling plaque iron for systemic use [27].

Direct confirmation that plaque iron accumulates through plaque rupture and hemorrhage has been lacking, partly due to the non-availability of an animal model in which to study this process [22]. We present observational data from ten symptomatic and fifteen asymptomatic human carotid artery plaques to support this proposed mechanism.

We have used synchrotron radiation induced X-ray fluorescence to map the distribution of iron within carotid plaque specimens. Our findings are consistent with data reported by others [13,28], demonstrating an inverse spatial relationship between iron and the mineralized portions of the plaque, which are rich in calcium and zinc. This finding supports the hypothesis that mineralized areas of plaque are load bearing, and confer mechanical stability [29]. This may account for the observation in clinical-pathological and radiographic studies that highly calcified carotid plaque tends to be more stable than plaque with little calcification [29–31].

We observe, within the subset of asymptomatic plaques, that diabetes is associated with significantly greater levels of calcium fluorescence. This is consistent with other data demonstrating diabetes as a risk factor for calcification of plaque [32]. This observation does not extend, however, to the group of symptomatic plaques. This is consistent with the preceding hypothesis, that calcified plaques are more stable. Symptomatic plaques are, by definition, unstable and represent a subset of plaques in which high degrees of calcification are less likely.

In symptomatic plaques, presenting with rupture and hemorrhage, we found iron within the thrombus. We also identified infiltration of the thrombus by macrophages, as early as five days after the onset of neurologic symptoms. Iron deposition in asymptomatic plaques is similarly associated with the presence of macrophages. This observation is consistent with macrophage mediated endocytosis of thrombus causing the iron to reside within the plaque after healing. The lack of PB staining of macrophage infiltrated thrombus in symptomatic plaques, and the presence of PB staining in asymptomatic plaques, is further consistent with transformation of heme iron to hemosiderin or ferritin within the macrophages. Interestingly, areas of PB staining are absent in symptomatic carotid plaques. We hypothesize that residual hemosiderin or ferritin within the plaque becomes obscured by fresh thrombus when the plaque ruptures. The lack of PB positive areas of plaque distant from the thrombus suggests that plaque rupture tends to occur in the location where previous iron deposits might have been located. This would be consistent with the hypothesis that iron in this area mediates the destabilization of the plaque.

Our data demonstrate that the abundance of iron fluorescence in symptomatic plaque is associated with the source patient’s LDL cholesterol. Patients with higher LDL cholesterol appeared to have a more extensive plaque hemorrhage, as determined by iron content, than patients with lower LDL cholesterol. The nature of this association is not apparent from our study. The mechanism by which elevated LDL cholesterol might promote more extensive plaque hemorrhage is unclear and it is possible that elevated LDL cholesterol is the consequence of the inflammation that drives plaque instability, rather than a direct cause.

The presence of subintimal iron deposits in the carotid plaques of patients lacking any history of prior ipsilateral neurologic symptoms, suggests that the process of plaque hemorrhage and healing can remain clinically silent when it occurs in the carotid arteries. This is consistent with findings in the coronary circulation, where plaques frequently undergo rupture without causing symptoms. It is possible that these carotid plaques do indeed cause ischemic injury, but no overt neurological symptoms, such as seen in silent strokes [33–38]. Despite not causing any identifiable symptoms, a silent stroke may still result in neurological damage and a significant increased risk in developing a major stroke in the future [39]. In addition to silent strokes, these healing events in asymptomatic plaques may form the basis to the eventual transition to symptomatic plaques. While the mechanism of this transition is not clear, we are currently investigating how the surrounding environment may play a role in altering both the activity of macrophages and local gene expression and thus transition the plaque to an unstable phenotype. It is hoped that with a better understanding of thrombosis formation and pathophysiology of atherosclerosis, that better diagnostic markers can be developed. Thus clinicians, with such a biomarker panel, will now be able to identify plaques prior to rupture and provide proactive treatment as opposed to reactive treatments, after neurological damage has occurred.

## Conclusions

Zinc and calcium co-localize in areas of plaque mineralization. Calcium is more abundant in asymptomatic carotid plaques from diabetics than non-diabetics. Iron localizes to areas of plaque, away from calcium and zinc. Heme iron is found in thrombus of symptomatic plaques, and is infiltrated by macrophages within days of a heralding neurologic event. The abundance of iron in the symptomatic plaque is associated with the patient’s LDL cholesterol level. Asymptomatic plaques also contain subintimal deposits of iron, but these are in the form of hemosiderin or ferritin and are also associated with the presence of macrophages. It is hypothesized that these macrophages play a key role in the creation of plaque instability and is the basis of our present studies.
